# Hospital and Home for Incurable Children

**Published:** 1902-05-17

**Authors:** 


### HOSPITAL AND HOME FOR INCURABLE CHILDREN.

### SPECIAL APPEAL.

Yet one more of these special appeals which seem to be one of the particular characteristics of the year 1902. But in this case, at any rate, the rebuilding of the home has become a matter of sheer necessity. The present building has for some time proved quite inadequate, and additional accommodation is earnestly required. It has, moreover, been discovered that the very large cracks existing in the old building will prevent its being improved or added to, and will necessitate the erection of a new building and the pulling down of the old. It is with this object in view that a special appeal, signed by H.R.H. the Duke of Connaught, the President of the home, and H.R.H. Princess Christian, one of the patrons, has been issued. The sum required is £20,000, and towards this over £5,000 has already been collected. The appeal has not yet been long enough before the public to allow of any conclusions being drawn as to its success or otherwise.

Annual Meeting of Governors.

The board-room on Tuesday, May 6th, was not uncomfortably crowded, but then, as one of the members of the committee pointed out, that only showed how implicit a trust was placed in the Committee of Management and how widespread was the confidence reposed in them. The chair was taken by the Rev. Canon Duckworth, D.D., and the report having been read by the hon. secretary, Mr. H. Sewell, the hon. treasurer, Mr. F. J. Lewis, proceeded to explain the accounts. It appeared from these that there was a deficit of £120 this year as against a deficit of £352 last year, so that on the whole the finances were in a fairly satisfactory condition, always excepting the heavy expenses that their rebuilding scheme entailed upon them. A concert that had recently been held on behalf of the home produced the gratifying sum of £215.

The formal business of the meeting was speedily despatched without any unnecessary waste of time, honorary officers being re-elected and the usual votes of thanks passed unanimously.

